# Metaphorical language and psychopathological symptoms: a case study of trauma victims’ metaphor use

**DOI:** 10.1186/s40359-023-01492-w

**Published:** 2024-02-01

**Authors:** Amy Han Qiu, Dennis Tay, Bernadette Watson

**Affiliations:** 1 Department of Philosophy, Linguistics, and Theory of Science, Faculty of Humanities, University of Gothenburg, Gothenburg, Sweden; 2 International Research Centre for the Advancement of Health Communication, Department of English and Communication, The Hong Kong Polytechnic University, Hong Kong SAR, China

**Keywords:** Metaphorical language, Symptom severity, Acute stress disorder (ASD), Psychopathological symptoms, Psychometric variables, Trauma metaphors

## Abstract

**Background:**

While clinical diagnosis of mental health issues focuses on factual details represented by literal language (e.g., the onset and process of the triggering event and duration of symptom), the relationship between metaphorical language and psychopathological experiences remains an intriguing question. Focusing on psychological trauma triggered by the 2019–2020 Hong Kong social unrest, this study explored the correlations between trauma victims’ quantitative metaphor usage patterns and their experience of specific Acute Stress Disorder (ASD) symptoms.

**Methods:**

Forty-six individuals with trauma exposure within 28 days were recruited through convenience sampling. Each completed a 20– to 30-minute semi-structured interview and filled out the Chinese version of the Stanford Acute Stress Reaction Questionnaire (SASRQ; 1). Metaphors in the interviews were identified using the discourse dynamic approach (2), and clinically interesting categories related to trauma and emotion expression, as revealed by previous literature, were sorted out. Standardized frequencies of the categories were correlated with participants’ SASRQ scores of five major ASD symptoms, and the correlational patterns were interpreted from a discourse analytic perspective.

**Results:**

The study reveals how metaphor usage patterns can reflect the speakers’ differentiated experiences of psychopathological symptoms. Compared with individuals who experienced less trauma, those more disturbed by the re-experiencing symptom were more inclined to use emotion-related metaphors and to metaphorize about the self and the self-society relationship. Individuals who experienced more severe anxiety and hyperarousal showed a heightened awareness of self-related issues and diminished attention to others. Those who suffered from more severe impairment in functioning produced more metaphors in the negative valence. Dissociation and avoidance, which were less experientially salient and intense than the others, were not significantly correlated with metaphor usage patterns.

**Conclusion:**

This study establishes symptom-level metaphor usage patterns as a previously overlooked but interesting avenue in trauma evaluation, treatment, and research. While the study is confined to a single context, it nevertheless reveals the potential for metaphor research findings to be incorporated as useful materials in psychology education and therapist training.

**Supplementary Information:**

The online version contains supplementary material available at 10.1186/s40359-023-01492-w.

## Introduction

Individuals who experienced or witnessed traumatic events may experience a mixture of overwhelming emotions, including but not limited to anxiety, anger, depression, and confusion. They may also suffer from a range of cognitive and physical disturbances, such as intrusive thoughts, flashbacks, and nightmares about the traumatic event, difficulty in sleeping and concentrating, and physical reactions such as headaches and nausea. According to the Diagnostic and Statistical Manual of Mental Disorders (DSM) outlined by the American Psychiatric Association (2013), these disturbances can be diagnosed as Acute Stress Disorder (ASD) within four weeks since trauma exposure and Post-traumatic Stress Disorder (PTSD) if the symptoms last longer than a month.

Trauma victims’ linguistic accounts of their personal experiences and subjective feelings constitute an important source of information for clinical assessment and therapeutic treatment [3, 4]. Among others, a particularly interesting linguistic phenomenon is metaphor, which is defined by some linguistic researchers as “the phenomenon whereby we talk and, potentially, think about something in terms of something else” [5, p.1]. Given that traumatic experiences can be intense, complex, and sometimes difficult to describe using literal language, trauma victims often seek to metaphorize their personal thoughts and feelings in terms of more vivid and widely shared experiences. For example, as noted by Wilson and Lindy [6, p.45], trauma victims might describe their perceived sense of deprivation as “I am *empty inside*” and the difficulty in engaging in meaningful interpersonal communication as “No one can *get close to* me”.

Systematic use of metaphors in different parts of the narratives or across therapeutic sessions often points to systematic patterns in thinking and feeling, such as conceptualizing the self as an empty container or in a secluded space. The patterns could provide valuable insights into clinically interesting experiences as well as the speakers’ personal and implicit ways of perceiving and understanding their own experiences [7, 8]. For this reason, systematic metaphor usage patterns are recognized by therapists as a useful tool for exploring trauma victims’ psychopathological experiences, promoting adaptive insights, and evaluating therapeutic progress [6–8].

Many mental health practitioners recognize the importance of attending to and exploring metaphors, but metaphors are usually not taken into account at the diagnostic stage, as the diagnosis is often prior to and separated from therapeutic treatment. In clinical scenarios, trauma-related symptoms and disorders are usually assessed based on diagnostic manuals such as the latest Diagnostic and Statistical Manual of Mental Disorders (DSM-V; [9]). As noted by Galatzer-Levy and Bryant [10], to establish reliability and ensure the validity of diagnoses, clinical practitioners prioritize universally shared, reliably observable aspects of the traumatic experience, such as the triggering event, the process and consequence of the event, and the duration of specific symptoms. Vague experiences that are not widely observable and documented are not considered in the diagnosis. As metaphors are often used by trauma victims to express elusive and idiosyncratic experiences, they are often left out as an irrelevant factor in trauma assessment and symptom-based research. Compared with metaphorical conceptualizations of trauma-related emotions and thoughts, we know much less about symptom-specific metaphor usage patterns. How metaphors are used by trauma victims with different symptom profiles and how the patterns vary across severities of symptoms remain interesting research questions.

In this paper, we present an exploratory case study on symptom-metaphor interactions in the context of the 2019–2020 Hong Kong social unrest. Metaphor usage patterns of 46 trauma victims were correlated with their experience of the five major symptoms of ASD, and the patterns were interpreted using genuine linguistic examples from a discourse analytical perspective. In what follows, we will summarize previous literature on trauma and metaphor use, introduce the research context and methods, and then present the statistical results and findings. Implications, limitations, and future directions will also be discussed.

## Literature review

Existing research has offered rich and detailed descriptions of trauma victims’ metaphor use in response to various traumatic events. Numerous studies have examined trauma metaphors in describing the emotional feelings, cognitive disturbances, and physical experiences associated with trauma (e.g., [11–16]). Some studies delved into metaphorical conceptualizations of trauma-related disorders, mostly PTSD (e.g., [6, 17–20]). The analyses focused primarily on metaphor topics and vehicle terms. Metaphor topics refer to the abstract, intangible issues that are being discussed, and metaphor vehicle terms are usually concrete, physical, and familiar experiences that offer a meaningful way of interpreting these topics [2]. Despite the limited sample sizes in many studies and the inclusion of individuals traumatized by different events, there is a general consensus that trauma victims often interpret their experiences in terms of basic bodily experiences, tangible objects in the physical and sociocultural context, and significant personal experiences. Commonly observed topics include the intrusion and long endurance of negative emotions, the altered sense of self, perceived changes in life, and the process of coping with stress and negative thoughts.

The way trauma victims use metaphors is closely associated with the intensity of traumatization. The differences in emotions, thoughts, and physical sensations can prompt the speakers to highlight or downplay specific aspects of their personal experiences or to organize and present their metaphorical ideas in particular ways [21].

Trauma victims’ choices of metaphor topics can vary remarkably with the degrees of traumatization. In an in-depth study of a PTSD client’s metaphor use, Wilson and Lindy [6] observed a clear shift in dominant metaphor topics as the client experienced reduced traumatic stress and gained positive insights. The client’s focus gradually shifted from (i) trauma-related physical and physiological experiences, emotional feelings, and thoughts to (ii) problem-solving cognitive activities for managing trauma-related thoughts, and eventually to (iii) the re-established continuous sense of self, reconnection to others, and re-integration into the broader sociocultural context. In one of the few studies that combined linguistic and psychometric data, Qiu and Tay [22] explored the relationship between trauma victims’ metaphor use and overall degrees of trauma, as indicated by the total scores of a trauma evaluation questionnaire. The findings showed that individuals with higher overall degrees of trauma generated more metaphors in describing personal emotions, thoughts, and self-identity compared to those who were less traumatized.

The variation of metaphor use across different trauma severities is also reflected by the speakers’ preference for vehicle terms. An in-depth analysis of interviews with seven individuals who developed PTSD due to different events, conducted by Costa and Steen [11], revealed a distinct shift from the trauma stage to the recovery stage. The shift was characterized by the transformation of vehicle groupings and the metaphor scenarios they built. Although both trauma and the subsequent recovery process were metaphorized using four basic vehicle groupings^1^, i.e., MOTION, MOVEMENT, CONTAINER, and SIGHT, the expressions fell into two contrasting metaphor scenarios. The traumatic stage was often described as a state of no MOTION or DOWNWARD MOVEMENT, a closed CONTAINER, and loss of SIGHT. In contrast, the recovery process was more frequently described as increased MOTION, UPWARD MOVEMENT, an open CONTAINER, and enhanced SIGHT. Similarly, Foley [12] reported transitions in metaphor vehicle groupings across recovery stages in a study of metaphors use by veterans with PTSD. As the veterans progressed in their recovery from PTSD, their choices of vehicle terms gradually shifted from the self being “controlled” or “dominated” by PTSD, to the self “surviving” PTSD, and eventually “embarking on a survivor’s mission”.

In addition to semantic aspects like vehicles and topics, less semantically meaningful aspects like emotional valence and psychological perspective [23] are also possible indicators of traumatization. Emotional valence refers to the speaker’s emotional tone or attitude conveyed by the metaphor, which may be negative, neutral, and positive. Psychological perspectives are the viewpoints from which a particular event or experience is described. There are two primary psychological perspectives: the field perspective, where the individual experiences the scene in the way it is from a first-person perspective, and the observer perspective, where the individual adopts an external standpoint, observing themselves in a more emotionally detached way “from the outside” [23, p.467]. In Qiu and Tay’s [22] study, individuals with higher degrees of trauma tended to use more metaphors in the negative valence. They also generated more self-inspecting metaphors from the observer perspective. In contrast, those who were less traumatized used a more proportionally balanced mix of negative, neutral, and positive metaphors and produced fewer self-inspecting metaphors.

While there has been abundant evidence of the relationship between metaphor use and trauma, much less attention was paid to the role of more specific symptoms. Wilson and Lindy [6, p.95] observed that the PTSD client often interpreted the experience of dissociation in terms of physical sensations of SPLITTING (e.g., “I am split apart” and “I am diffused and unglued”). In a study of war-traumatized refugees’ description of PTSD, Gušic et al. [24, p.546–547] reported similar expressions in describing dissociation, such as “my thoughts split” and “I don’t feel present sometimes, thoughts are not here”. In contrast, trauma victims’ experience of hypervigilance was often described as EXPLOSION using expressions like “I was about to explode” [6, p.69] and “being set off” [12, p.141]. In a recent study on metaphors in describing specific ASD symptoms, Qiu et al. [25] discovered that metaphors about different ASD symptoms had distinct image schematic groundings. For example, dissociation was mostly conceptualized in terms of DISABLEMENT and SPLITTING, and re-experiencing was often described using the CONTAINER and the COMPULSION schemas.

Existing studies show that diverse experiences of trauma can prompt the speakers to use metaphors in specific ways, and that certain aspects of metaphors can be particularly expressive indicators of the speakers’ psychopathological experience. These studies have shown interesting convergences in symptom-specific metaphors, which highlights the potential for metaphors to capture nuanced differences in symptom experiences. However, the studies have several limitations. Firstly, previous research often relied on linguistic data collected from PTSD patients during therapy or long after trauma exposure. Data on acute stress reactions, which are supposed to be collected within 28 days since trauma exposure, was scarce. Secondly, many previous studies focused on qualitative aspects of metaphor use, and some had small sample sizes or included individuals affected by different traumatic events. There have been very few large-scale investigations on individuals affected by the same traumatic event, especially the study of quantitative patterns that reflect general tendencies in metaphor use. Thirdly, while the studies extensively discussed how traumatization is reflected by metaphor use, they often relied solely on linguistic data and neglected the incorporation of psychometric data.

The present study explored the correlations between 46 trauma victims’ quantitative metaphor usage patterns and the severities of five ASD symptoms. The research was conducted during the 2019–2020 Hong Kong social unrest (see the next section for more details). Given its extended duration and the large number of trauma victims involved, the social unrest was regarded as an ideal context for conducting a large-scale study on ASD experiences. Based on linguistic data collected using semi-structured interviews and psychometric data collected using the Stanford Acute Stress Reaction Questionnaire (SASRQ; [1]), this paper addressed the following research question:

Do trauma victims’ use of clinically interesting metaphor variables correlate with the severities of ASD symptoms, i.e., dissociation, re-experiencing, avoidance, anxiety and hyperarousal, and impairment in functioning?

## Research background

The social unrest began in March 2019 in response to an extradition bill proposed by the government of the Hong Kong Special Administrative Region (HKSAR), which aims to facilitate the transfer of fugitives to jurisdictions not covered by existing laws, including the Chinese Mainland and Taiwan. Pro-democracy activists accused the government of undermining Hong Kong’s autonomy under the “one country, two systems” constitutional principle^2^. Initially, pro-democracy activists organized peaceful demonstrations such as sit-ins at the government headquarters. Starting from mid-2019, a series of more violent protests were launched, urging the government to address the five core demands proposed by the protesters^3^.

Protests escalated into violence and destruction and became increasingly uncontrollable by the end of 2019. From June to December 2019, extensive damage was inflicted on the city. A total of 740 sets of traffic lights were vandalized, and 52.8 km of railings along walkways and about 21,800 square meters of paving blocks on footpaths were removed [26]. Additionally, 85 Mass Transit Railway stations and 68 Light Railway stations were damaged. Protestors were reported to have used weapons such as metal bars, tear gas, catapults, and petrol bombs, and some resorted to physical and verbal assault as a means to “informally settle interpersonal conflicts” [27, p.621] with people who held different political opinions. In response, riot police employed tear gas, rubber bullets, bean bag rounds, and pistols to disperse radical protestors. This, in turn, triggered even more aggressive attacks from protesters [27, 28].

The social unrest resulted in heightened tension and polarization in the local community, giving rise to widespread anger, panic, and fear. According to a longitudinal study on Hong Kong residents’ mental health well-being [29], the weighted prevalence of depressive symptoms among adults during 2019–2020 was 37.4%, with a probable depression rate of 11.2%, surpassing levels observed in the past decade. The weighted prevalence of post-traumatic symptoms surged from 16.6 to 31.6% during the social unrest, and the rate of suspected PTSD was 12.8%, affecting approximately 810,000 people. The psychological impact of the social unrest was largely comparable to the influence of large-scale natural disasters, armed conflicts, or terrorist attacks [29].

This study focused specifically on traumatic experiences of mainland Chinese in Hong Kong. Individuals from this sociocultural group faced a particularly high risk of trauma, as some radical protesters directed violence and abuse specifically toward them, leading to significant distress and safety concerns [28, 30, 31]. The study employed a mixed-method design. Semi-structured interviews were conducted to explore the participants’ emotions and thoughts during the social unrest. Metaphors in the interviews were identified using the discourse dynamics approach [2] and further coded in terms of clinically interesting metaphor variables revealed by previous literature. The SASRQ was used to measure the participants’ acute stress reactions. Linguistic data and psychometric data were juxtaposed in correlation analyses, and the patterns were further interpreted from a discourse analytic perspective. More details about the research methods and data are provided in the next section.

## Methods and data

Data collection took place from mid to late December 2019, which was two to four weeks after a large-scale protest that had a wide-ranging influence on local residents’ lives. The sample consisted of 46 mainland Chinese (33 females and 13 males) who were either students or professionals working in Hong Kong during the social unrest. All participants met the DSM-V criteria for trauma exposure, which means they had either witnessed or experienced a highly distressing event, such as physical assault, violence to others, and destruction of buildings and public transportation^4^. Participants were recruited using convenience sampling via eight WeChat Groups established by university students, graduates, and staff who were originally from the mainland of China. All participants were native speakers of Mandarin Chinese, had received undergraduate education or above, and had an average age of 26.61 (SD = 4.52). None of them had sought mental health support for the traumatic event at the time of the study. Considering the intense social atmosphere, participants’ political stances were not solicited. However, all participants acknowledged that their lives and emotions had been greatly impacted by the social unrest and identified themselves as victims of the social unrest.

### Linguistic data

The participants were first invited to a semi-structured interview to discuss their emotional experiences during the social unrest. The interview questions and follow-up strategies were carefully developed in collaboration with a professional psychologist and counselling supervisor certified by the Chinese Psychological Association (CPA). At the time of the study, the therapist had more than 20 years of experience in crisis intervention and trauma therapy. All interviews were conducted by the first author, who had received professional training in psychotherapy and had 5years of experience in conducting mental health interviews. Considering the unstable social situation and safety concerns expressed by some interviewees, the interviews were conducted via audio calls.

Predetermined interview questions included “What was your strongest impression about the event?”, “How did the event affect your life and study/work?”, and “Can you describe your emotional experiences?”. Follow-up questions, mostly initiated with “why” and “how”, were used to elicit more specific descriptions of their traumatic experience. None of the questions targeted at specific ASD symptoms. All interviews were recorded and transcribed for further analysis. The total recording time was 16.67 hours, and the resulting transcripts contained 207,959 Chinese characters, with 177,981 contributed by the interviewees (averaging 3,869 per interview, SD = 1,751).

#### Metaphor identification

Metaphors in the interviews were identified using the discourse dynamics approach [2], which has been proven to be a clearly operationalized and reliable method for identifying metaphors in the mental health context [32]. Focusing on the interaction between language and multiple contextual factors (e.g., embodied experience, emotional feelings, and socio-cultural factors), this approach identifies metaphors from single lexical units to longer stretches of language like phrases and sentences.

Whether a stretch of language is metaphorical is determined based on its basic meaning and contextual meaning. The basic meaning refers to the contemporary meaning of the word or phrase in other communicative contexts, and the contextual meaning refers to the entity, relation, or attribute evoked by the current context [23]. If there is a “contrast or incongruity” between the two meanings and a meaning transfer that “enables that contextual meaning to be understood in terms of the basic meaning”, the stretch of language can be identified as a metaphor vehicle term, and what is being described by the vehicle is identified as the topic of metaphor [2, p.105].

To maximize the reliability of metaphor identification, a dictionary was used to establish a consistent criterion for determining basic meanings [2]. In this study, the Contemporary Chinese Dictionary (the 7th edition, henceforth CCD7) [33] was selected due to its extensive coverage of contemporary Chinese vocabulary and its clear differentiations between literal and figurative meanings. The identification is illustrated by the example below (vehicle term in bold type):

在我二十多年的人生里面, 应该是没有试过在短短的时间内情绪或者是心理状态会经历那么大的**起伏**。[More than 20 years have passed in my life, I have never experienced such great **rises and falls** in emotions or mental states within such a short period of time.]

According to the CCD7, the basic meaning of “起伏” is changes in vertical height. Its contextual meaning is the drastic emotional changes experienced by the speaker. Because the contextual meaning can be seen as an extension of the basic meaning, the phrase was identified as a metaphor vehicle term, and “emotional changes” was noted as the topic. Following this approach, 1,634 metaphorical vehicles were identified from the dataset (M = 35.52, SD = 25.62).

#### Metaphor variables and descriptive statistics

To extract the participants’ overall tendency in metaphor use, we calculated the density of vehicle terms per thousand characters for each participant. All metaphors were then coded in terms of clinically interesting variables revealed by previous literature, including conventionality, emotional valence, target categories, and perspectives. The operational definitions for the metaphor variables are given below, and examples for each variable category are presented in the appendix.

An important theoretical aspect that might be relevant to the metaphorization of trauma is *conventionality*, which refers to whether a metaphor is entrenched in everyday use by ordinary people for everyday communicative purposes [34]. Previous studies have reported interesting yet inconsistent findings regarding conventional and novel metaphors in emotional and cognitive activities. While some suggested that novel metaphors are more closely associated with intense emotional and cognitive processing than conventional metaphors [35–37], others, particularly in the context of mental health, highlighted the therapeutic relevance of both conventional and novel metaphors [38]. This variable was included in the present study to shed more light on the inconclusive debate. Following Steen et al. [39], whether a metaphor is conventional or novel was determined using the dictionary. If the contextual meaning of the metaphor vehicle was included in the CCD7 as one of the standard senses of the word, it was identified as a conventional metaphor; if not, it was identified as a novel metaphor.

The dataset was also coded in terms of *emotional valence*, a topic frequently discussed in the study of general trauma language [4, 40, 41] but rarely examined in the study of trauma metaphors. In this study, emotional valence was operationalized as the emotional tone that the speaker adopted when using metaphors. It was rated on a 3-point scale as negative, neutral, and positive. Metaphor vehicle terms that conveyed a pessimistic or disapproving attitude of speakers were categorized as “negative”, those without apparent emotional inclinations were coded as “neutral”, and those expressing an optimistic or approving attitude were coded as “positive”.

The concept of *target categories*, proposed by Kopp [42], aimed to capture the metaphoric structure of the interviewee's subjective reality. Originally, the taxonomy included six categories, i.e., SELF, OTHERS, SITUATIONS, SELF AND SELF, SELF AND OTHERS, and SELF AND SITUATION. Given the salience of social issues in the current dataset, an extended version of Kopp’s taxonomy was adopted following Qiu and Tay [22]. While preserving the other categories, the original “SITUATION” category was split into “PERSONAL SITUATION” and “SOCIAL SITUATION”, and the “SELF AND SITUATION” category was divided into “SELF AND PERSONAL SITUATION” and “SELF AND SOCIAL SITUATION”. Drawing from Lakoff’s [43] distinction between “subject” (i.e., the centre of consciousness, will, and judgment) and “self” (i.e., the rest of the self), cases where the speaker’s subject is conscious, compatible with the self, or has normal control over the self were coded as SELF. In contrast, those in which the two are incompatible, or either is unconscious or uncontrollable, were labeled as SELF AND SELF. Those depicting the self’s interactions with other people, personal life, and social situation were labeled accordingly.

The eight target categories can be further classified into the field and the observer *perspectives* [23], which are often examined by psychology studies as contrasting ways of narrating traumatic experiences (e.g., [44–46]). As introduced earlier, in the field perspective, individuals experience the scene in a self-immersed way, whereas in the observer perspective, individuals adopt a self-detached, or third-person standpoint in observing the self. In our study, target categories experienced with the speaker “inside” themselves (i.e., SELF, OTHERS, PERSONAL SITUATION, and SOCIAL SITUATION)were categorized as metaphorized from the field perspective, and categories that describe the relationship between the self and these four categories from an external viewpoint were considered from the observer perspective. As the two perspectives can be inferred based on the coding results of target categories, they were not included in the statistical analysis to reduce data redundancy; instead, they will serve as reference points in the subsequent discussion where relevant target categories are involved.

According to Fainsilber and Ortony [47] and Kövecses [21, 34], intense and salient subjective experiences such as trauma often bring about a heightened experience of specific physiological, emotional, and cognitive states. This heightened awareness can prompt more active use of these experiences as metaphor vehicle terms or topics. Based on previous research on trauma language and trauma metaphors (e.g., [4, 11, 12, 14]), we selected several vehicle groupings and discourse topics that are highly representative of trauma victims’ sensory, perceptual, and cognitive experiences. The vehicle groupings include SENSORY INFORMATION, WAR AND THREAT, SPACE AND SPATIAL RELATIONS, and PHYSICAL ACTIVITY, and the discourse topics include EMOTIONAL FEELINGS, SELF-REFERENCES, and THINKING AND UNDERSTANDING.

An overview of all metaphor variables and descriptive statistics for all variable categories is presented in Table 1. As the interviews varied substantially in length, participants’ metaphor usage patterns were summarised using the variables’ standardized frequencies per thousand characters rather than raw frequencies.

#### Interrater reliability

As metaphor identification using the discourse dynamics approach accepts indeterminate boundaries, it is not always possible to evaluate the interrater reliability of coding using quantitative measures. To maximize the reliability of coding, the CCD7 was used to assist the determination of basic meanings. Ambiguous cases were settled through discussion with experienced metaphor researchers and the therapist who was involved in the project.

Since determining metaphor variable categories involves making categorical decisions on fixed analytical units, the reliability of coding could be systematically checked using quantitative interrater reliability measures. Following Bolognesi et al. [48], a novice rater of a non-linguistic background was invited for reliability checks. After going through the whole dataset, the rater was trained using the abovementioned coding schemes. The two raters worked independently on 15% of the data, which were randomly selected using Excel. Krippendorff’s alpha was then calculated to assess inter-rater agreement. Krippendorff’s alphas for the coding of emotional valence, target category, vehicle groupings, and discourse topics were 0.741, 0.865, 0.697, and 0.721, respectively. All of these values were greater than the minimum acceptable reliability (i.e., α = 0.667) suggested by Krippendorff [49]. Since the determination of conventionality relied on dictionary meanings, no additional check was needed.

**Table 1:** An overview of all metaphor variables and descriptive statistics

Metaphor variables		Mean of density (per thousand Chinese characters)
Conventionality	Novel Conventional	3.82 (SD = 2.09) 5.10 (SD = 2.41)
Emotional valence	Negative Neutral Positive	4.47 (SD = 1.98) 3.89 (SD = 2.33) 0.56 (SD = 0.64)
Target categories	SELF OTHERS PERSONAL SITUATION SOCIAL SITUATION	1.93 (SD = 1.31) 1.31 (SD = 1.64) 1.02 (SD = 1.02) 1.06 (SD = 0.98)
	SELF AND SELF SELF AND OTHERS SELF AND PERSONAL SITUATION SELF AND SOCIAL SITUATION	0.98 (SD = 1.25) 0.44 (SD = 0.67) 0.61 (SD = 0.53) 1.56 (SD = 1.24)
Trauma-related vehicle groupings	SENSORY INFORMATION PHYSICAL ACTIVITY WAR AND THREAT SPACE AND SPATIAL RELATIONS	1.93 (SD = 1.14) 2.02 (SD = 1.28) 0.48 (SD = 0.63) 0.76 (SD = 0.65)
Trauma-related discourse topics	EMOTIONAL FEELINGS SELF-REFERENCES THINKING AND UNDERSTANDING	3.10 (SD = 1.89) 0.46 (SD = 0.73) 1.49 (SD = 1.28)

### Psychometric questionnaire and descriptive statistics

To recap, this study investigated the link between trauma victims’ metaphor use and symptom severities. Shortly after the interview, the participants had their degrees of trauma measured using the Chinese version of the SASRQ [50]. The SASRQ is a 30-item self-report that measures the subject’s experience of five specific symptoms:

**Dissociation** refers to the alterations in the perception and awareness of self, others, and the surrounding environment.

**Re-experiencing** reflects the extent to which the subject relives the memories and feelings that are related to the traumatic event.

**Avoidance** describes the subject’s tendency to avoid traumatic-related stimuli, such as thoughts, activities, people, feelings, etc.

**Anxiety and hyperarousal** refers to the individual’s increased anxiety, sensitivity, and physiological arousal to external stimuli.

**Impairment in functioning** reflects the extent to which the subject’s physical, cognitive, and social functioning is affected.

Each questionnaire item is rated on a 6-point scale from 0 to 5, ranging from “not experienced” to “very often experienced”. We can either calculate the total score to assess the subject’s overall degrees of trauma or extract the symptom scores to examine the severityies of specific symptoms^5^.

In this paper, we focused on the severities of specific symptoms. The severity of a symptom is indicated by the corresponding subscale score, which is the sum of all item scores that measure the same symptom [52]. A high score on a specific symptom suggests that the participant is particularly disturbed by that symptom, and vice versa. Both the original and the translated questionnaire have demonstrated good reliability and validity [1, 53, 54]. The internal consistency of participants’ ratings and descriptive statistics are summarized in Table 2. There were no missing data in the dataset.

**Table 2:** Descriptive statistics and internal consistency of SASRQ scores

Variable	Number of items	Cronbach’s alpha	Descriptive Statistics	
			Mean/total score	Standard Deviation
Dissociation	10	0.837	9.91/50	7.07
Re-experiencing	6	0.773	5.98/30	4.57
Avoidance	6	0.886	9.70/30	7.38
Anxiety and hyperarousal	6	0.826	10.37/30	5.78
Impairment in functioning	2	0.635	3.13/10	2.37

### Combining linguistic and psychometric data

To investigate the relationships between the participants’ metaphor use and psychopathological experiences, standardized frequencies of the metaphor variables were correlated with severities of the five major ASD symptoms. According to the Central Limit Theorem, sampling distribution in a sample of 30 or above tends to be normal, regardless of the actual distribution of data. Therefore, the parametric measure of correlation, i.e., Pearson’s *r*, was used. Following previous research on trauma language (e.g., [40, 55, 56]), *p* = .05 was used as the threshold for statistical significance. The analyses were conducted using Jamovi 2.0.0.0. Significant patterns were then interpreted from a discourse analytic perspective to provide a contextualized understanding of the patterns in the participants’ actual language use.

## Results and discussion

### Correlation analyses

Significant correlations between trauma victims’ metaphor use and severities of the five ASD symptoms are summarized in Table 3. As the study aimed to identify symptom-related metaphor usage patterns, only metaphor variables that showed significant results were included.

**Table 3:** Significant correlations between metaphor use and the experience of symptoms

Metaphor Variables	Psychometric variables				
	Dissociation	Re-experiencing	Avoidance	Anxiety and hyperarousal	Impairment in functioning
Negative metaphors	*n.s*	*r =* .292, *p =* .049*	*n.s*	*n.s*	*r* = .462, *p* = .001**
OTHERS	*n.s*	*n.s*	*n.s*	*r=*-.319 *p =* .030*	*n.s*
SELF AND SELF	*n.s*	*r =* .385, *p =* .008**	*n.s*	*r =* .319 *p =* .031*	*n.s*
SELF AND SOCIAL SITUATION	*n.s*	*r =* .308, *p =* .037*	*n.s*	*n.s*	*n.s*
Emotional feelings and processes	*n.s*	*r =* .300, *p =* .043*	*n.s*	*n.s*	*n.s*
Self-references	*n.s*	*n.s*	*n.s*	*r =* .325, *p =* .028*	*n.s*

(Note: *p*-values less than 0.05 are indicated as * and those less than 0.01 as **. Non-significant results are indicated as *n.s*)

Results show that trauma victims’ use of the selected metaphor variables was significantly correlated with the severities of three ASD symptoms, including re-experiencing, anxiety and hyperarousal, and impairment in functioning. Severities of re-experiencing were positively and significantly correlated with two emotion-related variables, i.e., the negative valence and emotional feelings and processes. These two symptoms also showed positive and significant correlations with SELF AND SELF and SELF AND SOCIAL SITUATION. Anxiety and hyperarousal scores had significant and positive correlations with two self-focused variables, i.e., SELF AND SELF and self-references, while showing negative correlations with OTHERS. Severities of impairment in functioning were significantly related to metaphors in the negative valence. In contrast, dissociation and avoidance did not show significant correlations with any metaphor variables (all *p*s > 0.05).

### Discourse analysis

In this section, significant correlations are illustrated using genuine linguistic examples and discussed in the order of symptoms. It is important to note that the examples are not necessarily direct accounts of the symptoms (refer to 25 for metaphors in describing ASD symptoms). Instead, the examples demonstrate how trauma victims, under the influence of specific symptoms, can use metaphors to highlight or downplay certain aspects of their experience.

#### Re-experiencing

As the severity of re-experiencing increases, trauma victims become more inclined to metaphorize their emotional experiences during the traumatic event. Compared with those less disturbed by re-experiencing, individuals with more severe symptoms tend to use more negative metaphors. They were also more inclined to metaphorize the perceived incongruence within the self and the interactions between the self and the broader social situation.

The tendencies are illustrated by example (1). The example was generated by an interviewee whose re-experiencing score was 2.19 SD above the mean score of 5.98 (16 out of 30 points). It is a negative metaphor about EMOTIONAL FEELINGS AND PROCESSES, with a special emphasis on the incongruency between different aspects of the self (i.e., SELF AND SELF):

 (1)那个时候我的心情也是处于一种**撕扯**吧。一方面, 我要像老师说的那样理性地看待世界, 然后并且用我自己所学的知识来理解当下社会发生的一切。 但是另一方面, 我**站在一个无辜卷入的被害、受害者的角度**, 我还是没有办法去理性地去认识他们(施暴者), 然后还是会很感情用事地觉得他们到底在干什么? [At that time, my emotions were **tearing me apart**. On the one hand, I need to look at the world in a rational way, just like what my supervisor said, and use what I have learned to understand all that was happening in this society. But on the other hand, I am **standing in the position of an innocent victim, who was involuntarily drawn into this event**, I still can’t understand them (the radical protesters) in a rational way. I was very emotional and kept asking myself what on earth are they doing?]

Two metaphor vehicle terms were used to describe the speaker’s negative emotional feelings during the peak of the social unrest. In the first metaphor vehicle term, the confrontation between two different emotional and thought processes was conceptualized as the speaker being physically torn into two different parts. One part stands for the speaker’s rational thinking, which enables her to get rid of all personal feelings and make sense of the social unrest in a detached and objective way, and the other part represents the emotional aspect of the self, which is totally disconnected from the rational side of the self. The latter aspect is further elaborated by the second metaphor vehicle, which describes the self as an unlucky “victim” who was “involuntarily drawn into this event”.

The tendency for individuals with severe re-experiencing symptoms to focus on emotions and express negative feelings is compatible with previous psychological research findings, which underscores negative emotional response as a key component of re-experiencing, alongside trauma-related sensory impressions and other substantive details of the traumatic event [40, 57–61]. According to the DMS-V, individuals exposed to trauma-related cues can experience intense or prolonged psychological distress as if the traumatic event is happening again. The positive and significant correlation between re-experiencing scores and emotion-related metaphors is also consistent with Fainsilber and Ortony’s [47] findings that descriptions of intense emotional experiences contain more emotion-related metaphors than accounts of milder emotions. As intense emotions are presumably more vivid and remarkable as compared to milder experiences, individuals may feel a more pressing need to provide detailed descriptions of their emotional states, which, in turn, leads to increased use of metaphorical expressions.

Compared with individuals who had less severe re-experiencing, those more disturbed by the symptom also generated more metaphors about SELF AND SELF and SELF AND SOCIAL SITUATION, which tap directly into the speakers’ emotions and thoughts about the social unrest.

The first metaphor vehicle term in example (1) is a typical instance of SELF AND SELF metaphors. In contrast to SELF metaphors, which provide a self-immersed perspective on the speaker’s thoughts and feelings, this target category opens a self-inspecting perspective on the speaker’s emotions and thought processes. It captures the inconsistency between different emotions, thoughts, and the individual’s pre- and post-trauma sense of self, which is a crucial cognitive challenge faced by all trauma victims when processing their experiences [62]. The increased use of SELF AND SELF metaphors reflects the imminent psychological need for trauma victims to reconcile the conflicts between their emotions and rational thinking and to make sense of the clash between their personal and social identities.

Topics about the current social situation are also frequently observed in trauma victims’ conceptualization of their experience. While SOCIAL SITUATION depicts trauma victims’ perception and understanding of the societal status from a first-person perspective, SELF AND SOCIAL SITUATION places greater emphasis on how the speakers’ everyday life and future development are connected to, and further influenced by the social unrest. It captures the participants’ struggles in finding their roles in the rapidly changing social situation, which was, at the time of the social unrest, the most immediate and greatest psychological challenge faced by mainland Chinese immigrants.

The use of SELF AND SOCIAL SITUATION metaphors by high scorers on the re-experiencing subscale is illustrated by example (2). This speaker’s re-experiencing score was 1.31 SD above the sample mean (12 out of 30 points).

 (2) 对于未来你可能计划得很好, 然后你有一些你的打算, 但是**在这个时代的洪流面前, 你就直接被碾过去了**, 一点办法都没有。[Maybe you have done a lot of preparations for your future, and you might have formed some of your own plans, but **when faced with this flood torrent of history, you just get crushed over**, and there’s nothing you can do.]

The interviewee was a fresh graduate who planned to find a job and settle down in Hong Kong. However, the unfriendly atmosphere created by the social unrest, especially the protesters’ destruction of the interviewee’s laboratory in the university and the interpersonal conflicts arising from differing political views, made her realize that Hong Kong may not be a good place for mainland Chinese to stay. In this expression, the social unrest is interpreted as an event of great historical significance. A vivid metaphor scenario is constructed to express her idiosyncratic understanding of how ordinary people in Hong Kong were involuntarily affected by rapid social changes: the social unrest and its impact on the Hong Kong society are interpreted as a gigantic and irresistible “flood torrent”, which can lead to permanent and irreversible changes to the surrounding environment, and the impact of the social unrest on the speaker’s study and future development is conceptualized as the speaker being physically “crushed over” by the “torrent” and straying away from the original position.

The positive and significant correlation between SELF AND SOCIAL SITUATION and re-experiencing is consistent with the observation that trauma victims under the influence of re-experiencing tend to concentrate on aspects of trauma that had the largest psychological impact on the individual [63, 64]. While previous research focused almost exclusively on concrete autobiographical details that are directly pertinent to the traumatic event (e.g., trauma victims’ sensory impressions of trauma-related images, sounds, smells, physical experiences, and substantive details such as the onset, process, and development of the traumatic event; see [59–61, 64]), the present study shows that abstract thinking elicited by concrete traumatic experiences, reflected by metaphor use, can also be active among individuals with severe re-experiencing symptoms.

#### Anxiety and hyperarousal

The severities of anxiety and hyperarousal were significantly correlated with increased use of self-related metaphors. Compared with individuals less disturbed by anxiety and hyperarousal, those experiencing more severe symptoms were more inclined to use SELF AND SELF metaphors to describe the conflicts between their thoughts and emotions (see example 1). Moreover, this trauma population also generated more self-referential metaphors to account for the perceived changes in their self-identities.

Below is an example of self-referential metaphors, produced by an interviewee who got 18 points for anxiety and hyperarousal, which is 1.32 SD above the sample mean.

(3) 当时就会有一些很焦虑的心理状态, 第二个就是暴躁, ……, 因为会觉得自己是无辜的, 自己是**受了无妄之灾**的。 [At that time, I often felt a lot of anxieties in my mind, and the second feeling is angry, …, because I would think that I am innocent, I am a** victim of an unexpected disaster**.]

In contrast to example (1), which focuses on the opposition between the rational subject and the emotional self, this metaphor describes how the self, as a unified entity, experienced a sudden and unexpected change in identity. The impact of the traumatic event is conceptualized as an integral part of the self. Based on the conceptualization of the traumatic event as an unforeseen natural disaster, the speaker, under the influence of anxiety and hyperarousal, perceived herself as taking on an additional metaphorical identity, i.e., an innocent victim of a sudden disaster.

An interesting pattern that contrasts with the high scorers’ inclination toward self-focus is the negative and significant correlation between anxiety and hyperarousal scores and the density of OTHERS metaphors. This suggests that individuals who experienced more severe anxiety and hyperarousal were less inclined to metaphorize other people’s behaviors, feelings, and thoughts, in contrast to low scorers on this subscale. A close examination of OTHERS metaphors in the dataset reveals that the topics include people in the trauma victim’s close interpersonal circle, such as family, friends, and colleagues, or people involved in the social unrest, such as the protesters, Hong Kong residents in general, and news media. Examples (4) and (5), which address the two topics, are provided to illustrate this pattern:

(4) 我身边同事有黄有蓝, ……, 的确他们自己这么说自己, 我就引用一下。但是我心中没有蓝黄之说, 就是**没有颜色之分**。因为每个人都是**七色**, 都是**彩虹**。[Some of my colleagues are yellow and some are blue, …, they indeed refer to themselves in this way, I’m just citing their words. But in my mind, the distinction between blue and yellow does not exist, I mean there’s **no difference of color** at all. Because everyone has **seven different colors**, everyone is a **rainbow**.]

(5)我们日常都是本来就局限在学校这个小圈子里, … 学校毕竟是个挺净土的一个地方, 之前愤怒也是因为你怎么**把手伸到**学校这块地方来了。[Our daily life has always been restricted within this small circle of the university, …The university is, after all, like a pure land. The reason why I felt angry at that time is that how can you l**ay your hands on** the university.]

Example (4) was provided by an interviewee who got only 3 points on the anxiety and hyperarousal subscale (1.28 SD lower than the sample mean). In Hong Kong, the two major political camps in Hong Kong are traditionally symbolized by different colors: the pro-establishment camp is often represented by blue, and the pro-democracy camp by yellow. Due to the social unrest, Hong Kong residents became increasingly attentive to their own and other individuals’ political stances. Those who identified with the blue and the yellow camps frequently found themselves in opposition, and sometimes in direct confrontations. In this example, the speaker expresses her disagreement with the blue-versus-yellow division through an extension of the POLITICAL STANCE IS COLOR metaphor. Instead of categorizing people using binary labels of blue and yellow, the speaker describes the complexity of human nature as a person having “seven different colors” and being a “rainbow”.

Example (5) was produced by a participant who did not report evident anxiety and hyperarousal, as measured by the SASRQ. A metaphor describing radical protesters’ acts was used right after a PERSONAL SITUATION metaphor (i.e., the university as a pure land) to express the speaker’s disapproval of the protesters’ vandalism of her university campus. The university, which was expected to stay clear from political activities, is compared to a sacred “pure land”. The protesters’ forceful imposition of their political views on the university staff and students is conceptualized as a physical act of “laying their hands” on a precious and revered object.

The others-focused tendency identified in low-scorers was rarely found in individuals who experienced severe anxiety and hyperarousal. This can be a natural consequence of the self-focused tendency that accompanies anxiety and hyperarousal [65, 66]. While the symptom prompted the individual to allocate more cognitive resources to the processing of self-related thoughts and feelings, there would be correspondingly less attention available for matters considered “external” or less relevant to the self.

#### Impairment in functioning

Individuals with higher scores on the impairment in functioning subscale showed a significantly stronger tendency to use metaphors with the negative valence. Example (1), which was presented earlier, is illustrative.

According to Jellestad et al. [67], post-traumatic functional impairment manifests as perceived difficulties in accomplishing a wide range of real-world tasks, including but not limited to everyday self-care, managing domestic life, handling interpersonal interactions, and coping with general life tasks and demands. According to Ni et al. [29], trauma victims of the social unrest perceived difficulties in maintaining their previous life routines, concentrating on work and studies, and preserving previous interpersonal interactions with friends and family. Individuals more severely affected by this symptom would experience heightened frustration and a greater sense of losing control when dealing with such functioning difficulties. This emotional response would then drive them to generate more negative metaphors in expressing their thoughts and feelings after trauma.

Although metaphorical thinking about abstract aspects of the traumatic event may not be inherent components of intrusive memories, the expressions still offer valuable insights for understanding the speaker’s immediate psychopathological experience. While previous studies have shown that individuals with different overall degrees of trauma used metaphors in distinct ways [22], this study revealed metaphor usage patterns that are related to more specific symptoms of ASD. It was found that trauma victims with differentiated experiences of ASD symptoms showed distinct preferences toward different metaphor variables. As the severity of trauma and symptoms changes, the attention to or preference for the metaphor variables also varies.

A particularly interesting finding is that trauma victims who experienced more re-experiencing and anxiety and hyperarousal used more self-focused target categories than the less traumatized. When the findings are considered in relation to the field and observer [23], more intriguing patterns emerge. Recall that SELF AND SELF and SELF AND SOCIAL SITUATION metaphors, both are from the observer perspective, were significantly correlated with severities of re-experiencing, whereas their counterparts from the field perspective (i.e., SELF and SOCIAL SITUATION) were not. A similar pattern was found in the relationship between SELF AND SELF metaphors and the experience of anxiety and hyperarousal. In other words, those more affected by these symptoms showed a stronger preference for using metaphors from the observer perspective.

McIssac and Eich’s [45] study on perspective-taking in traumatic memories suggested that narrations in the field perspective contained richer details about the individual’s affective reactions, somatic sensations, and psychological states during the traumatic event, whereas narrations in the observer perspective provided more factual details about the traumatic situation and less emotional content. Meanwhile, numerous studies on perspective-taking in trauma narratives showed that patients with PTSD tend to adopt an observer perspective when recalling trauma-related memories [45, 68]. At first glance, the two sets of findings might seem contradictory, as PTSD patients, who are typically overwhelmed by negative emotions, appear to prefer the less emotion-laden perspective. Clinical psychologists propose that the contradiction can be explained by trauma victims’ tendency to maintain a psychological distance from extremely distressing memories [46]. While re-experiencing the traumatic event can be intrusive and overwhelming, adopting an external, self-observing perspective enables the individual to reprocess the traumatic experience from a safe psychological distance, preventing them from being overwhelmed again. This interpretation is supported by Robinson and Swanson’s [44] experimental studies on perspective changes. According to their findings, as the field perspective shifts to the observer perspective, the intensity of emotion experienced tends to decrease as the perspective shifts from the field to the observer perspective. In other words, the preference for the observer perspective over the field perspective in metaphor use might reflect the speakers’ efforts to adopt a detached standing point when re-processing difficult traumatic memories.

Another intriguing finding is that statistically significant correlations were primarily related to emotional valence, target categories, and discourse topics, rather than conventionality and vehicle terms. A previous study on trauma metaphors indicated that conventional and novel metaphors in different topics and perspectives can differ remarkably in emotional valence [22]. It is therefore possible that the relationship between conventionality and psychopathological experiences varies across different types of metaphors. Future research could explore this possibility by breaking down conventional and novel metaphors into different emotional valence, topics, and perspectives, or examine whether the role played by trauma-related variables differs across the two types of metaphors.

The fact that no vehicle term was significantly correlated with symptom scores does not necessarily mean that the latter is not relevant to trauma victims’ psychopathological experiences. One possible reason is that vehicle groupings in this study were identified in a top-down manner based on the findings of previous trauma and metaphor research. While the coding method is helpful for exploring the relevance of clinically interesting metaphor variables to trauma evaluation, it may not fully capture the nuances of the current research context. To better account for the relationships between trauma victims’ experience of ASD symptoms and their tendencies in using vehicle terms, future research could consider using systematic, bottom-up coding methods.

We also observe that the number of significant correlations differed across the five ASD symptoms. Re-experiencing and anxiety and hyperarousal showed the most robust correlations with metaphorical language, each significantly correlated with three metaphor variables; impairment in functioning scores were significantly correlated with one metaphor variable; dissociation and avoidance were not significantly related to any metaphor variables. The difference is likely related to the nature of the symptoms. Re-experiencing, anxiety and hyperarousal, and impairment in functioning entail heightened subjective experiences, including emotional feelings, thoughts, bodily sensations, responses to trauma-related cues, and reflections on details about the traumatic event and its impact on the self. As the symptom severity goes up, these subjective experiences become more intense and vivid, which will then compel trauma victims to provide more elaborate accounts of their feelings [47]. In contrast, dissociation and avoidance are characterized by reduced awareness of the traumatic experience and trauma-related emotions and thoughts. Compared with re-experiencing, anxiety and hyperarousal, and impairment in functioning, the two symptoms are much less experientially prominent. Given that milder emotional experiences are less likely to trigger increased use of metaphors than intense feelings [47], it is understandable that experiences of the latter two symptoms were less strongly correlated with quantitative metaphor usage patterns.

## Conclusion

While trauma victims’ metaphors have rarely been considered a relevant factor in clinical evaluation and diagnosis, this study shows that systematic metaphor usage patterns can be expressive indicators of ASD symptoms, establishing symptom-level metaphor usage patterns as a previously overlooked but interesting research avenue. Compared with the less traumatized, individuals more affected by re-experiencing generated more metaphors in the negative valence. They also used more metaphors in describing their emotions, inner conflicts, and their relationship with the social situation. Individuals who experienced more severe anxiety and hyperarousal showed a heightened self-focused tendency and reduced attention to others. Those who experienced more severe impairment in functioning produced more metaphors in the negative valence. The patterns are largely consistent with the clinical manifestations of corresponding symptoms, which demonstrates the potential for metaphor variables to reflect nuanced psychopathological experiences that have distinct physiological, emotional, and cognitive characteristics.

The study illustrates the advantage of combining linguistic and psychometric data in exploring trauma victims’ self-accounts. It also highlights the need for clinical practitioners to pay closer attention to trauma victims’ metaphor use, especially general tendencies reflected by quantitative patterns. While this study is limited to a single context, it reveals the possibility for quantitative metaphor usage patterns to capture clinically interesting experiences and the potential for metaphor-based analysis to assist in clinical explorations of psychopathological symptoms. Furthermore, typical patterns and linguistic examples could be incorporated as useful teaching or supporting materials in therapist training and education on trauma treatment and metaphor-based protocols (e.g., [42, 69–72]). As metaphors often capture the speakers’ personal feelings and thoughts, the analysis of symptom-related metaphor variables can help to explore trauma victims’ idiosyncratic experience of the symptom of clinical interest. Developing a keener sense of metaphor use would also be helpful for fostering a “patient-centered” perspective [73, 74] in clinical practices.

Key limitations in this study should be acknowledged. Firstly, this study is based on a sample collected using convenience sampling, and the participants’ demographic characteristics were relatively homogeneous. To better capture the psychological impact of large-scale traumatic events, it would have been ideal to use stratified random sampling that could reflect the demographic landscape of the targeted population. Secondly, the generalizability of findings might be undermined by potential confirmation bias. For example, individuals suffering from avoidance symptoms might be less inclined to sign up for the study than those with other symptoms. Subsequent research could consider collecting a larger sample with more diversified demographic features and traumatic backgrounds to validate the findings of this study. Thirdly, owing to the unexpected nature of the social unrest, this study only focused on participants’ spontaneous metaphor use right after the traumatic event. The longitudinal evolution of metaphors across different stages of trauma remains to be explored. As the present traumatic event was influenced by complex social, cultural, and political factors, it would also be interesting to explore how the use of metaphors is shaped by the speakers’ ideological beliefs. Finally, it is worth mentioning that *p* = .05 is not the fixed threshold for statistical significance. While this decision is helpful for exploratory research to derive clear-cut conclusions about theoretically interesting and potentially clinically relevant patterns, a *p*-value only provides “a crude orientation regarding the probable realness of specific group differences” [75, p.815]. Future researchers could consider interpreting *p*-values as a continuous measure and supplement the results with more descriptive statistics (e.g., confidence intervals and data distribution) to provide a more comprehensive account of their data [76].

This study suggests several promising avenues for future research. While our study focused specifically on trauma victims’ metaphor use, we did not explore whether the patterns are unique to metaphorical language or also apply to literal accounts of trauma. Subsequent research could explore potential differences and similarities between metaphorical and non-metaphorical expressions. Additionally, previous research and our study have highlighted the roles of ASD and PTSD in shaping language use, which raises a further question: does language use differ between trauma victims whose ASD progressed to PTSD and those who soon recovered from ASD? Addressing this question would require a longitudinal study with a larger group of trauma victims. Finally, while this study focused on bivariate relationships between single clinical symptoms and metaphor use, future research could explore how multiple symptoms or the number of clinically present symptoms may interact with metaphor use.

### Electronic supplementary material

Below is the link to the electronic supplementary material.

**Supplementary Material 1: Appendix 1.** Linguistic examples for all metaphor variables

## Data Availability

The datasets generated and/or analysed during the current study are not publicly available due to ethical reasons but can be made available from the corresponding author on reasonable request.

## Footnotes

1 Following the conventions used by the discourse dynamics approach, emergent themes of metaphor vehicle terms and target topics in English will be represented using capital letters.

2 “One country, two systems” describes the governance of Hong Kong and Macau, which became Special Administrative Regions of the People’s Republic of China in 1997 and 1999, respectively. It indicates that while the Mainland of China adopts the socialist system with Chinese characteristics, the two special administrative regions, under the principle of “one China”, could retain their own governmental systems and legal, economic and financial affairs, independent from those of the mainland.

3 The five demands include the withdrawal of the extradition bill, universal suffrage, inquiry into alleged police brutality, amnesty for arrested protestors, and retraction of the classification of protesters as rioters.

4 Although some residents may have experienced negative emotions due to social media and interpersonal tensions, their experiences did not involve immediate life threats or physical injuries. Consequently, they did not meet the criteria for trauma as defined by the DSM-V [9].

5 Alternatively, the ratings could also be converted to dichotomous data to account for the clinical presence of specific ASD symptoms (refer to 49 for more details). More details regarding clinically present symptoms in the current context can be found in Qiu et al. [25], and a comprehensive overview of participants’ psychological wellbeing is available in Qiu [51].
