# Supplementary material for: Metaphorical language and psychopathological symptoms: a case study of trauma victims’ metaphor use
Source: BMC Psychol. 2024 Feb 1;12:57. doi: 10.1186/s40359-023-01492-w (PMC10835999; doi:10.1186/s40359-023-01492-w)
Supplement: Supplementary file 1 — Supplementary Material 1: Appendix 1. Linguistic examples for all metaphor variables [file 40359_2023_1492_MOESM1_ESM.docx]

Appendix 1. Linguistic examples for all metaphor variables

| Variables | Categories | Linguistic Example |
| --- | --- | --- |
| Conventionality | Novel | 生活中仿佛那些五彩斑斓的东西不见了。[It’s like all those colorful things in my life had disappeared from sight.] |
|  | Conventional | 一开始有情绪波动的时候，我很快就调整过来，但是后面就是一种挥之不去的感觉。[At the beginning, when I felt some emotional fluctuations, I could adjust myself very quickly, but it then became very difficult to wave it off.] |
| Emotional valence | Negative | 我自己甚至都可能会意识到我是不是就像（进入）一个黑洞一样，被负面情绪吸进去了。 [Even I myself realized, that I might be, just like (in) a black hole, absorbed by negative emotions.] |
|  | Neutral | 这个事情，我感觉啊，就是个小小插曲。 [This event, I feel, is just like a small episode.] |
|  | Positive | 可能我也是这个时候容易被感动了，突然间就会觉得好像感受到了希望之火慢慢地燃烧起来。[Maybe I was particularly sentimental at that time, suddenly I felt that the fire of hope was starting to burn.] |
| Target category | SELF | 我觉得可能就是一种比较无奈或者说无力的感觉。[What I felt is probably helplessness, or say, lack of strength.] |
|  | OTHERS | 学校毕竟是个挺净土的一个地方，之前愤怒也是因为你怎么把手伸到学校这块地方来了。[The university is, after all, like a pure land. The reason why I felt angry is they laid their hands on this place.] |
|  | PERSONAL SITUATION | 生活中仿佛那些五彩斑斓的东西不见了。[It’s like all those colorful things in my life had disappeared from sight.] |
|  | SOCIAL SITUATION | 那会儿(学校名)已经就成战场了，然后失控状态。 [At that time (university name) has already become a battlefield, and lost control.] |

(to be continued on the next page)

(continued from the previous page)

| Target category | SELF AND SELF | 通过那段经历来学会一些辩证思考, …, 把自己从当时的环境脱离开来, 作为一个旁观者去分析。[Through my experience of the event I learned to think in a dialectical way, …, get myself detached from the environment, take on the role of an onlooker when analyzing everything.] |
| --- | --- | --- |
|  | SELF AND OTHERS | 我也不冒犯你，但我就远离你们就好。[I don’t want to offend you, but I will just stay away from you.] |
|  | SELF AND PERSONAL SITUATION | 尤其是停课之后，就把我整个这一年的这种努力和充实感都给稀释掉了。[Especially when all classes were suspended, it just diluted all the efforts I made and the sense of achievement.] |
|  | SELF AND SOCIAL SITUATION | 以前觉得还可以，就是我们不谈，我们不说，我们置身事外，但是现在就发现不行，就是政治已经糊在你脸上了，就是你跑都跑不掉了。[I thought it was ok, we could stay out of it as long as we don’t talk about it. But now it doesn’t work anymore. Politics has been pasted to your face now; you can’t run away from it anyway.] |
| Trauma-related  Vehicle groupings | Sensory information | 有点烦躁，有点焦躁，也有点像是有点透不过气的感觉。[I felt a bit agitated, a bit irritated, or difficult to breathe.] |
|  | War and threat | 但是作为第一线、在主战场来说，我觉得主要令我感觉很不爽、很愤怒的点，可能还是对个人的影响。[Being at the frontline, at the main battlefield, what annoys me the most is how the event had affected my own life.] |
|  | Space and spatial relations | 在我二十多年的人生里面，应该是没有试过在短短的时间内情绪或者是心理状态会经历那么大的起伏。[More than 20 years have passed in my life, I have never experienced such great rises and falls in emotions or mental states within such a short period of time.] |
|  | Physical activity | 一开始有情绪波动的时候，我很快就调整过来，但是后面就是一种挥之不去的感觉。[At the beginning, when I felt some emotional fluctuations, I could adjust myself very quickly, but it then became very difficult to wave it off.] |

(to be continued on the next page)

(continued from the previous page)

| Trauma-related  Discourse topics | Emotional feelings | 我自己甚至都可能会意识到我是不是就像（进入）一个黑洞一样，被负面情绪吸进去了。 [Even I myself realized, that I might be, just like (in) a black hole, absorbed by negative emotions.] |
| --- | --- | --- |
|  | Self-reference | 会觉得自己是无辜的，自己是受了无妄之灾的。 [Because I think I am innocent, I am a victim of an unexpected disaster.] |
|  | Thinking and understanding | 就是试图抛掉我所处的环境，把这个事情放到更大的格局上来看。[I tried to get rid of the environment I am now in, and look at this event in a bigger backdrop.] |

(end of table)
